# Effect of zirconia surface topography on epithelial attachment following diode laser-assisted crown lengthening: A controlled split-mouth animal study

**DOI:** 10.1016/j.jobcr.2026.101456

**Published:** 2026-04-23

**Authors:** Mohamed H. Helal, Mohamed Yehia Abdelfattah, Mohamed T. Elhalawani, Moustafa N. Aboushelib

**Affiliations:** a Oral Medicine, Periodontology, Oral Diagnosis and Radiology Department, Faculty of Dentistry, Tanta University, Tanta, Egypt; b Oral Biology Department, Faculty of Dentistry, Beni-Suef University, Egypt; c Department of Basic Dental Sciences, Faculty of Dentistry, Ibn Sina University for Medical Sciences, Amman, Jordan; d Conservative Dentistry Department, Faculty of Dentistry, Alexandria University, Egypt; e Prosthodontics Department, Faculty of Dental Medicine, Alamein International University, Egypt; f Dental Biomaterials Department, Faculty of Dentistry, Alexandria University, Egypt

**Keywords:** Crown lengthening, Zirconia surface roughness, Epithelial attachment, Soft tissue healing, Diode laser, Histomorphometry

## Abstract

**Objective:**

To evaluate the effect of zirconia surface topography on epithelial attachment and peri-restorative soft tissue healing following diode laser-assisted crown lengthening.

**Materials and methods:**

A controlled split-mouth experimental study was conducted in 12 Beagle dogs. Bilateral mandibular sites received CAD/CAM zirconia restorations with either glazed or micro-roughened cervical surfaces. Crown lengthening was performed using a diode laser, followed by full thickness mucoperiosteal flap elevation and osteoplasty. Clinical parameters and histomorphometric analysis were assessed at 2 and 4 weeks postoperatively.

**Results:**

Micro-roughened zirconia surfaces demonstrated significantly enhanced epithelial attachment, improved collagen organization, and superior soft-tissue adaptation compared with glazed surfaces (*P* < 0.05). At 2 weeks, inflammatory infiltration and disorganized collagen were observed. By 4 weeks, marked tissue maturation and epithelial integration were evident, particularly in the micro-roughened group.

**Conclusion:**

Zirconia surface topography significantly influences peri-restorative soft tissue healing. Micro-roughened surfaces promote superior epithelial attachment and tissue integration following laser-assisted crown lengthening.

## Graphical abstract

A controlled split-mouth experimental study in Beagle dogs evaluated the effect of zirconia surface topography on epithelial attachment following diode laser-assisted crown lengthening. Micro-roughened zirconia surfaces demonstrated significantly enhanced epithelial attachment, improved collagen organization, and superior soft tissue adaptation compared with glazed surfaces during early healing (P < 0.05).

**Figure:**
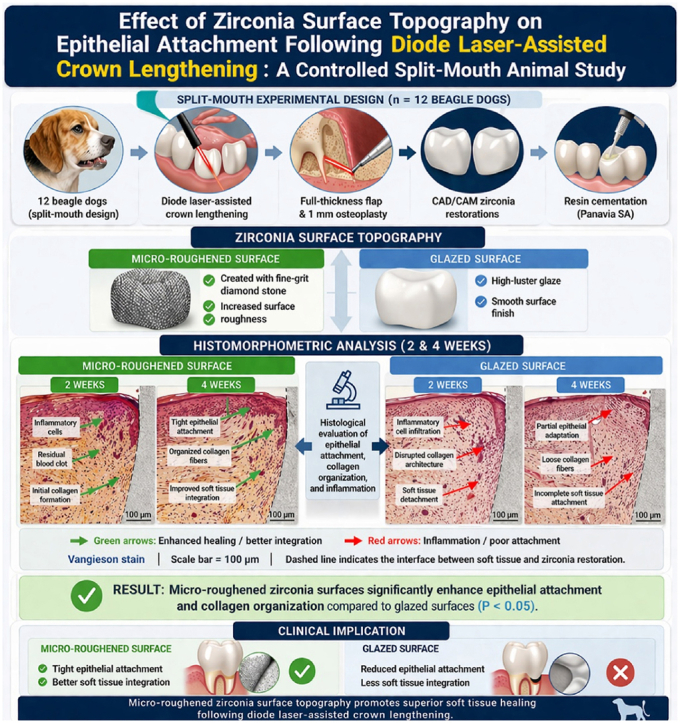

## 1 Introduction

Healthy epithelial attachment, a key factor governing the long-term health of the periodontium around fixed restorations, could be affected by several factors, including marginal adaptation, surface finish, and contouring of the inserted restoration, the location of its margins in relation to the alveolar bone crest, as well as proper soft tissue management during tooth preparation and impression procedures.^1^ In this context, the success of crown lengthening surgery, one of the most common procedures in the field of periodontics, remains of utmost importance in cases requiring the adjustment or restoration of the biological width.^2^^,^^3^ The technique could be performed using a surgical blade or different types of soft tissue laser irradiation devices, among which the diode laser is one of the most commonly used for this procedure.^4^^,^^5^

Diode laser, having a shorter wavelength, penetrates the epithelium and soft tissue with a depth of 2-6 mm. It is characterized by having a deep penetration and coagulation depth of the irradiated soft tissue, causing better hemostasis and improving post-surgical healing. It also causes less inflammation and edema around the cut tissue.^6^^,^^7^ The surface layer of the wound produced by cutting, called char or coagulum, protects the wound from friction and bacterial action for 2-3 days post-operatively until it eventually disintegrates.^8^ However, laser irradiation of soft tissue requires mastering of power adjustments and hand strokes to prevent excessive soft tissue damage around the cutting laser tip.^9^

Following surgical crown lengthening, a blood clot is formed, and initial inflammation is followed by gradual remodeling and re-organization of soft tissue architecture, ending in re-formation of the epithelial lining of the gingival sulcus and re-establishment of the attachment epithelium on the newly exposed root surface. Presence of temporary restorations^10^^,^^11^ and proper surface finish of the final restorations are known to influence the final shape and architecture of soft tissue surrounding the cervical margin of the restorations.^12^^,^^13^ Currently, limited data exist in the dental literature about the relationship between attachment epithelium and surface finish of all-ceramic restoration following crown lengthening procedures. However, data reported in the literature suggest that different materials and surface topographies affect human gingival fibroblast morphology, proliferation, and gene expression.^14^ Surface topography of restorative materials is a critical determinant of cellular behavior, influencing protein adsorption, fibroblast adhesion, and extracellular matrix organization. While smooth surfaces may reduce plaque accumulation, micro-roughened surfaces have been shown to enhance soft tissue attachment and integration. However, limited evidence exists regarding the influence of zirconia surface characteristics on epithelial attachment following crown lengthening procedures. Therefore, the present animal study aimed to evaluate epithelial attachment against high translucency zirconia following a soft tissue laser crown lengthening procedure. The null hypothesis of the current study was that the surface finish of the restoration would not influence soft tissue healing and attachment after a crown lengthening procedure.

## 2 Materials and methods

This study was designed as a split-mouth experimental model, in which bilateral mandibular sites within the same animal received different surface treatments (glazed vs. micro-roughened), allowing intra-animal comparison and minimizing biological variability. Sample size calculation was performed based on detecting a small effect size with a statistical power of 0.95 and a significance level (α) of 0.05, following established guidelines for animal studies (Charan and Kantharia, 2013).^15^ 12 male beagle dogs, 2 years in age, were enrolled in this study based on sample size calculations. All dogs were kept under hygienic conditions in an animal house and provided with a standard diet and ad libitum. All dogs were properly nourished, housed, and evaluated by a professional vet at the Faculty of Agriculture, Alexandria University. Veterinary care was provided by a specialist with necessary vitamin supplements and periodical health check-ups. The protocol of the experiment was approved by the ethics committee of the Faculty of Dentistry, Tanta University, Egypt number #R- OMPDR-9-23- 3052.

### 2.1 Crown lengthening protocol

All animals received general anesthesia using 2% isoflurane (Forene; Abbot Scandinavia, Solana, Sweden) supplied with proper intubation. Soft tissue laser crown lengthening was performed bilaterally on the mandibular premolar/molar region of each dog to remove 2 mm of the attached gingiva using a diode laser apparatus (Epic pro, Biolase, USA), which emitted irradiation at a wavelength of 980 nm at a frequency up to 10000 Hz using a peak pulse setting at 7-W irradiation power. A full thick mucoperiosteal flap was carefully elevated to expose the underlying alveolar bone, followed by osteoplasty: 1 mm of crestal bone was removed and recontoured using a hard tissue laser (Waterlase iPlus 2, Biolase) to re-establish the biological width. The flap was repositioned to its original position to allow optimal healing. Immediate temporalization after crown lengthening was performed using an injectable polymethyl-methacrylate resin (Protemp 4; 3M ESPE, St Paul, MN, USA) and a preformed rubber index.^10^

### 2.2 Fabrication of all-ceramic restorations

Mandibular molars were prepared to receive all-ceramic restorations, ensuring 0.8 mm axial reduction and 0.9 mm occlusal clearance. Impressions were made using an additional silicone medium consistency material, and the obtained casts were laser-scanned. Jaw relation was recorded using an additional silicone bite recording material (OBite; DMG Chemical Pharmaceutical Factory GmbH, Hamburg, Germany). High translucency zirconia CAD/CAM milling blocks were selected for fabrication of the final restorations (ZirCAD, HT A3; Ivoclar Vivadent, Schaan, Liechtenstein). After sintering, the cervical 2 mm of the restorations received one of the following surface treatments: either a modified machined surface using a fine-grit diamond stone or by applying addition-glaze using the manufacturer's recommendations. Fabricated restorations were cemented using a resin cement under conscious sedation (Panavia SA, Kuraray, Osaka, Japan).

### 2.3 Periodontal indices

The following parameters were evaluated for each experimental tooth before and after laser crown lengthening surgery: position of the gingival margin, probing depth, modified gingival index (MGI), ^16^ plaque accumulation using Turesky-Gilmore-Glickman plaque index (TGGPI) ^17^; presence or absence of bleeding on probing (BOP; 1 = present and 0 = absent). All measurements were made at six sites (mid-facial, mid-lingual, mesial-facial, mesial-lingual, distal-facial, distal-lingual), and the average of the six readings was obtained.

### 2.4 Histomorphometry evaluation

After 2 and 4 weeks of surgery time, tissue blocks were fixed in 4 % paraformaldehyde in 0.1 M phosphate buffer, followed by ascending dehydration in ethyl alcohol. Dehydrated blocks were immersed in methyl-methacrylate resin (Technovit® 7200 VLC; Kulzer, Hanau, Germany). By using a precision cutting machine (Exakt®; Apparatebau, Norderstedt, Germany), the blocks were cut in a buccal-lingual plane, and the most central section was collected from every specimen. The obtained central sections were reduced to a thickness of approximately 100 μm using rotating silicon carbide paper, then stained using van Gieson stain. The relative proportions of the tissue occupied by collagen (Co), fibroblasts (Fi), vascular structures (V), mononuclear leukocytes, and polymorph nuclear leukocytes, macrophages, lymphocytes, and plasma cells were evaluated using a light microscope (Leica DM-RBE®; Leica, Heidelberg, Germany) equipped with digital camera (Q-500 MC®; Leica, Heidelberg, Germany). Images were analyzed using image analysis software (BZ analyzer software; Keyence, Itasca, IL, USA). Vertical and horizontal marginal adaptation were also assessed in addition to the height of epithelial attachment.

### 2.5 Statistical analysis

All data were analyzed using computer software (SPSS 22, SPSS inc, IBM, Chicago, USA). Students' t-test was used for data analysis, including two surface treatments and two assessment times. Based on the sample size (n = 12), a selected small effect size, and significance value determinant (ά = 0.05), the power of the test was 0.95. Data was checked for normality, and outliers were detected and examined.

## 3 Results

**Fig. 1:**
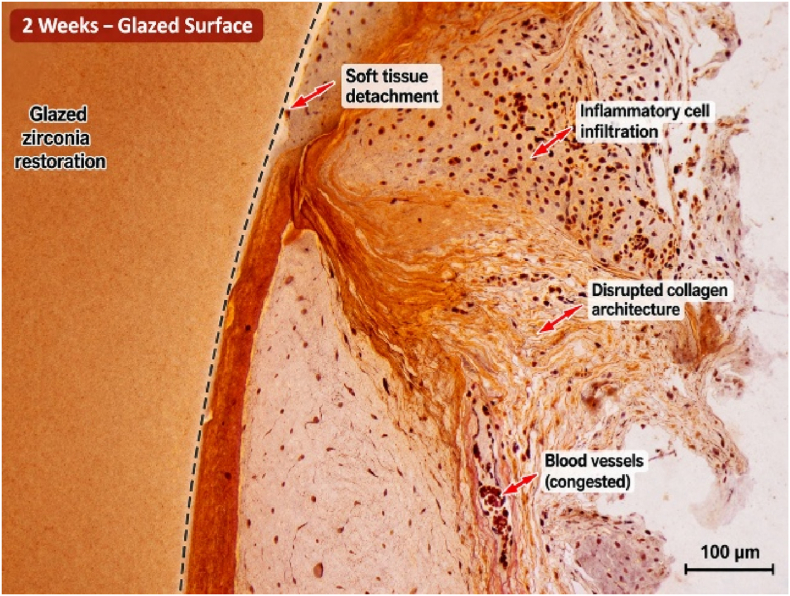
Histological section at 2 weeks demonstrating inflammatory cell infiltration, disrupted collagen architecture, and soft tissue detachment adjacent to glazed zirconia restoration. (Scale bar = 100 μm).

**Fig. 2:**
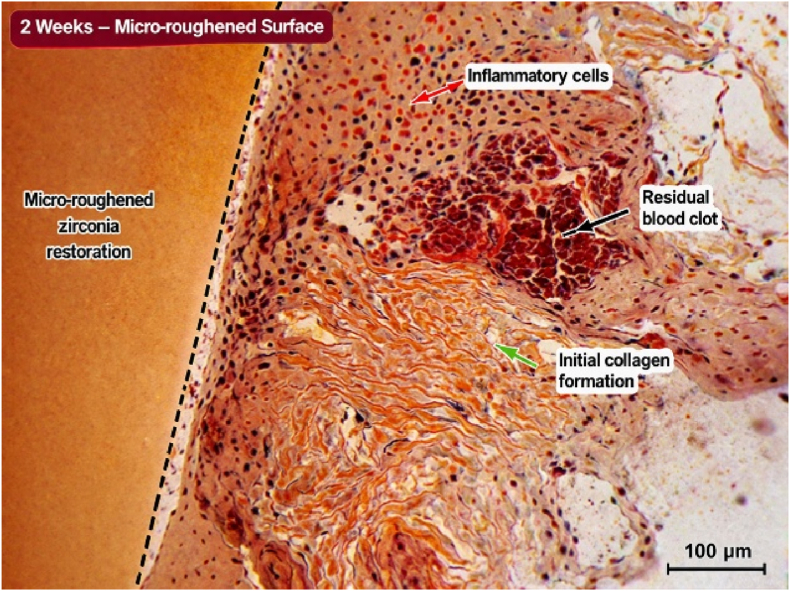
Histological section at 2 weeks demonstrating early healing response with inflammatory infiltration and residual blood clot adjacent to micro-roughened zirconia restoration. (Scale bar = 100 μm).

**Fig. 3:**
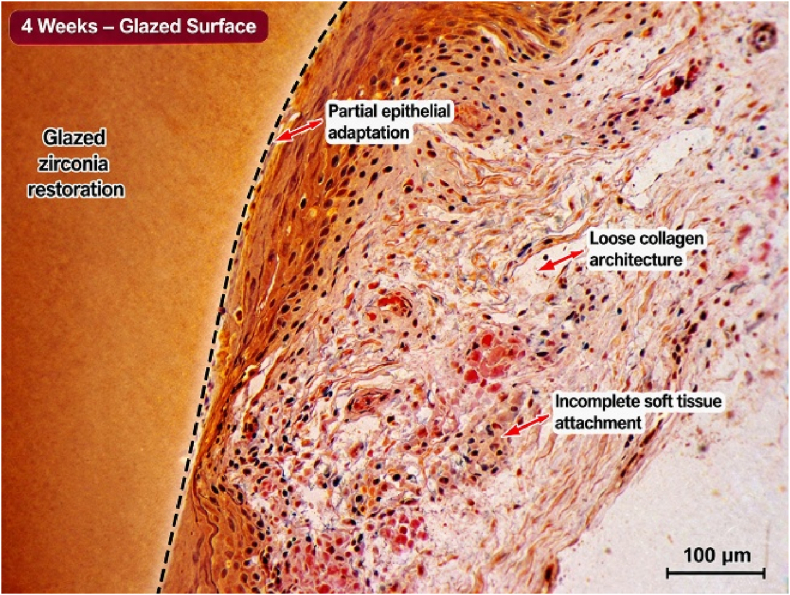
Histological section at 4 weeks showing partial tissue maturation and limited epithelial adaptation around glazed zirconia restoration. (Scale bar = 100 μm).

**Fig. 4:**
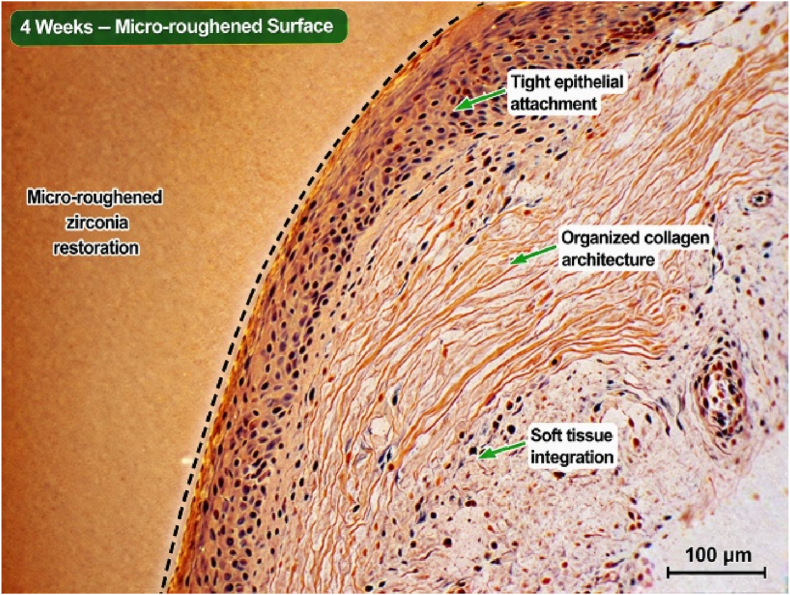
Histological section at 4 weeks demonstrating organized collagen fibers, enhanced epithelial attachment, and improved soft tissue integration around micro-roughened zirconia restoration. (Scale bar = 100 μm).

Histological examination at 2-week intervals revealed the presence of inflammatory cellular infiltration and the presence of soft tissue lacerations in the areas treated with soft tissue laser. There were also observed gaps between soft tissue margins and the finish line of the cemented restorations. Remnants of blood clots in addition to disrupted collagen fibers were also observed at the gingival sulcus (Fig. 1, Fig. 2). After 4 weeks, tissue fibers were more organized and adapted to micro-rough restorations with complete recovery of inflammatory signs as observed by the disappearance of inflammatory cells and the early-formed blood clot (Fig. 3. Wider tissue gaps were observed for glazed restorations (Fig. 4).

No statistically significant differences were observed at 2 weeks (t = 6.7, *P* > 0.05). However, Significant differences were observed at 4 weeks (t = 14.7, *P* < 0.05), favoring the micro-roughened surface. Promoted better tissue healing and remodeling, Table 1.

**Table 1:** Gingival health parameters according to surface treatment and time intervals.

Surface Treatment	Time Point	Plaque Index (score)	Bleeding on Probing (score)	Keratinized Tissue Width (mm)	Probing Depth (mm)	Modified Gingival Index (score)
Glazed	Baseline	1.67 (0.41)	1.67 (0.52)	5.50 (0.45)	3.42 (0.80)	0.50 (0.84)
	2 weeks	4.23 (1.47)	1.33 (0.52)	3.75 (0.27)	2.50 (0.45)	1.50 (0.55)
	4 weeks	5.14 (1.22)	1.67 (0.52)	3.83 (0.26)	2.90 (0.40)	0.50 (0.55)
Micro-rough	Baseline	1.00 (0.55)	1.83 (0.41)	5.33 (0.52)	3.33 (0.60)	0.33 (0.52)
	2 weeks	3.33 (0.82)	1.33 (0.52)	3.92 (0.20)	2.33 (0.41)	1.33 (0.52)
	4 weeks	4.92 (1.46)	2.00 (0.01)	4.17 (0.26)	2.08 (0.20)	0.33 (0.52)

Values are presented as mean (standard deviation). Probing depth and keratinized tissue width are expressed in millimeters (mm). Statistical significance was set at *P* < 0.05.

## 4 Discussion

Placement of immediate provisional restorations after crown lengthening surgeries has been recommended in the literature, as it enhances healing of the gingival tissues and attached epithelium.^18^ Surface topography plays a critical role in modulating cellular responses. Micro-roughened surfaces enhance protein adsorption and increase surface wettability, thereby promoting fibroblast adhesion and extracellular matrix formation.^19^^,^^20^ This may explain the improved epithelial attachment observed in the present study.

It is also advised that the final restorations should be placed at least 3 months after the crown lengthening procedure, for the soft tissue margin to reach its final position.^21^ Soft tissue management after crown lengthening could be complicated by bleeding and soft tissue laceration; however, once properly done, it will allow for proper soft tissue contouring and will allow controlled remodeling against the margins of the provisional, which is a great advantage for pink esthetics.^18^

In this study, a temporary pre-shaped restoration was immediately inserted after crown lengthening surgery to protect soft tissue margins and to act as an early shaping template for the required soft tissue contour.^10^ A temporary restoration will eliminate the need to cover the wound with a perio-pack.^18^ To standardize all the specimens and to observe the effect of the surface roughness on healing of the soft tissues, the final restoration was inserted within the first 48 h to allow for completion of soft tissue healing and final remodeling and re-contouring of collagen fibers. This could be achieved due to the use of the proper laser irradiation for the procedure, which resulted in a dry field and allowed early placement of restorations.^21^^,^^22^ This technique will prevent future relapse of soft tissue margins with time, and soft tissue healing will not be further disrupted, plus it will gain sufficient protection provided by the deflection ridges of the restoration.^19^

Early inflammatory responses were observed after two weeks of surgery, but were completely resolved after 4 weeks.^23^ Despite that, reformation of attachment epithelium was observed as early as 4 weeks; final remodeling and recontouring of soft tissue margins is expected to last even longer.^24^^,^^25^ Data analysis revealed that soft tissue healing was significantly enhanced against a micro-rough ceramic surface compared to a glazed one. After four weeks of the crown lengthening procedure, inflammation had already subsided, and soft tissue was already in the early remodeling phase, with evidence of epithelium lining the gingival sulcus, in addition to reformation of epithelial attachment on a micro-roughened surface. The physicochemical properties, mainly wettability and hydrophilic/hydrophobic nature, of a biomaterial affect cell adhesion by influencing protein adsorption and extracellular matrix constitution.^26^^,^^27^

In the current study, the modified gingival index scores fall within the normal score range of 0.5 – 2.4 reported by multiple studies, whether before or after the crown lengthening procedures 28, 29, 30. Those scores increased significantly 2 weeks after the procedure; however, by the 4th week, the inflammation showed signs of subsiding. This is in agreement with clinical trials that observed stability in the crown-lengthening level of the gingival margin after the same period of time.^31^

Because the presence of plaque on a tooth surface is considered a primary causative factor for the development of gingival disease, it was important to observe the plaque retention on both surfaces.^30^ It was observed that the plaque index and bleeding on probing index in the current study were within previously reported limits.^28^

Superior healing quality on micro-roughened surface was also associated with lower horizontal marginal gaps, indicating soft tissue proximity to micro-roughened surface, while higher vertical marginal adaptation values indicated success of the soft tissue margin to maintain its relative position after completion of the crown lengthening procedure. This might be due to the increase in wettability, caused by the micro-roughening, which influences protein adsorption onto the biomaterial and allows strong adhesion of epithelial cells onto it.^26^^,^^32^^,^^33^

On the contrary, lower vertical marginal adaptation values associated with glazed ceramic surfaces indicate that a mirror-like surface does not promote soft tissue healing and remodeling after cutting and damaging the attachment epithelium. Glazed dental restorations provide a smooth hydrophilic surface, which restricts cellular adhesion to their surface.^20^ It is also postulated that a mirror-like surface interferes with cell attachment, secretion of attachment proteins, and thus may delay or interrupt the formation of attachment of epithelium.^20^^,^^32^

It has been shown in the literature that fibroblasts tend to follow the direction of the grinding marks present on the surface of restorations and tend to orient their long axis parallel to these grinding marks.^34^^,^^35^ In a study by Gómez-Florit et al.,^36^ Fibroblasts were shown to attach to a titanium/zirconium alloy in large numbers after 2 days of cell culture. On the machined surface, the cells were aligned and exhibited parallel thick actin stress fibers; however, on a polished surface, the cells exhibited a rounded morphology without a clear orientation and lacked stress fibers.^34^^,^^36^ The alignment of the fibroblasts on micromachined surfaces might be caused by the mechanical stresses created by the presence of micro-grooves.^34^^,^^35^ It has to be mentioned that both materials use an almost identical surface glaze with similar chemical and physical properties. Future studies with longer follow-up periods and clinical human models are recommended to validate these findings and further investigate the long-term stability of peri-restorative soft tissues.

## 5 Strengths of the study

This study utilized a controlled split-mouth design, minimizing inter-animal variability. Standardized surgical and prosthetic protocols, along with histomorphometric analysis, enabled precise evaluation of epithelial attachment and soft tissue healing.

## 6 Limitations of the study

This study was conducted on an animal model, which may limit direct clinical extrapolation. The follow-up period was limited to early healing phases, and only two surface treatments were evaluated.

## 7 Conclusions

Within the limitations of this study, zirconia surface topography significantly influences peri-restorative soft tissue healing. Micro-roughened zirconia surfaces promoted superior epithelial attachment, enhanced collagen organization, and improved marginal adaptation compared with glazed surfaces during early healing following diode laser-assisted crown lengthening.

## Ethical approval

The Study protocol and methodology were conducted following the ethical principles for medical research involving animals and human subjects, approved by the Research Ethics Committee (REC) of Tanta University, specifying conditions and constraints for conducting and publishing studies involving animal models (approval no***# R-OMPDR-9-23-3052***), and followed the ARRIVE guideline. REC is organized and operated according to Enhancing Research Ethics Committees in Egypt, Guidelines for Standard Operating Procedures, Monitor 2006, Guidelines of the Declaration of Helsinki, International Conference of Harmonization ICH, and United States Code of Federal Regulations.

## Funding

This research was supported through self-funding by the authors.

## Declaration of competing interest

The authors declare that they have no known competing financial interests or personal relationships that could have appeared to influence the work reported in this paper.

## Data Availability

The row data of this research could be obtained from the authors upon request.
